# Primary pulmonary diffuse large B-cell lymphoma with multiple ground-glass nodules as the primary manifestation: A case report

**DOI:** 10.1097/MD.0000000000023501

**Published:** 2020-12-11

**Authors:** Qi Wang, He Yan, Rangrang Wang, Chunyan Li, Wei Li, Yanling Xu, Zhenzhong Su, Jie Zhang

**Affiliations:** a Department of Respiratory and Critical Care Medicine, The Second Affiliated Hospital of Jilin University; b Department of Emergency Medicine, The First Hospital of Jilin University, Changchun, Jilin, China.

**Keywords:** diffuse large β-cell lymphoma, ground-glass opacity, primary pulmonary lymphoma, pulmonary nodules

## Abstract

**Introduction:**

Primary pulmonary lymphoma (PPL) is a rare extranodal lymphoma. Only 5% to 20% of patients suffering from PPL have diffuse large β-cell lymphoma (DLBCL), and their chest computed tomography (CT) findings show single- or double-lung patchy or flocculated shadows, isolated or multifocal nodules, or masses. In this research paper, we report an older woman having multiple ground-glass nodules, who was eventually diagnosed with primary pulmonary diffuse large β-cell lymphoma (PPDLBCL).

**Patient concerns:**

A 69-year-old woman suffering from cough was admitted to the Second Hospital of Jilin University.

**Diagnoses:**

A chest CT scan showed multiple ground-glass nodules. She had received 2 weeks of antibiotic treatment, but the multiple ground-glass nodules were still present. Lung biopsy was performed by tracheoscopy, which showed non-Hodgkin diffuse large β-cell lymphoma.

**Interventions:**

The patient received R-CHOP-21 chemotherapy.

**Outcomes:**

The multiple ground-glass nodules were absorbed.

**Conclusion:**

The current study shows that spotting multiple ground-glass nodules in the lungs is a clear indication of the presence of PPDLBCL. It is important to spread awareness of PPDLBCL, which needs timely diagnosis and management.

## 1 Introduction

Primary pulmonary lymphoma (PPL) is a rare extranodal lymphoma. It usually manifests as a mucosa-associated lymphoid tissue (MALT); however, sometimes, it presents as diffusely large β-cell lymphoma (DLBCL).^[1]^ There are often no specific clinical and imaging changes that occur due to its clinical manifestations, and it is clinically diagnosed as pulmonary lymphoproliferative lesions or other tumors. Here we report a case of primary pulmonary diffuse large β-cell lymphoma (PPDLBCL), which was significant because of its particularly rare imaging manifestation that showed rapidly growing multiple changes of ground-glass nodules (GGNs). In addition, we briefly review the literature related to PPDLBCL.

## 2 Case presentation

**Figure 1:**
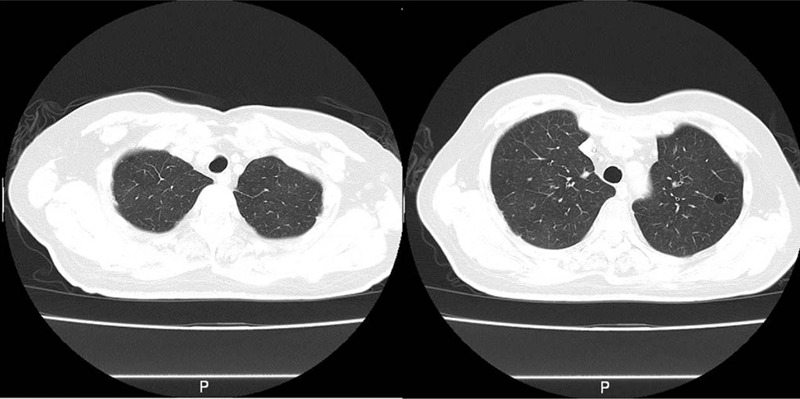
The initial stage of onset. Chest CT shows no obvious abnormalities. CT = computed tomography.

**Figure 2:**
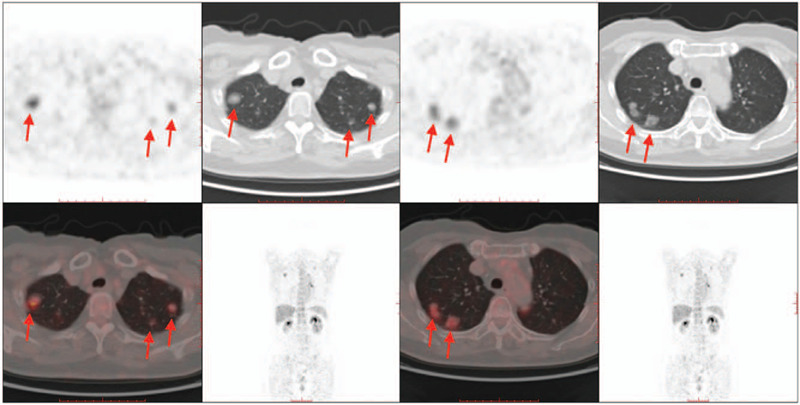
PET-CT demonstrated multiple lung-grinding glass nodules with a partial metabolic increase.

**Figure 3:**
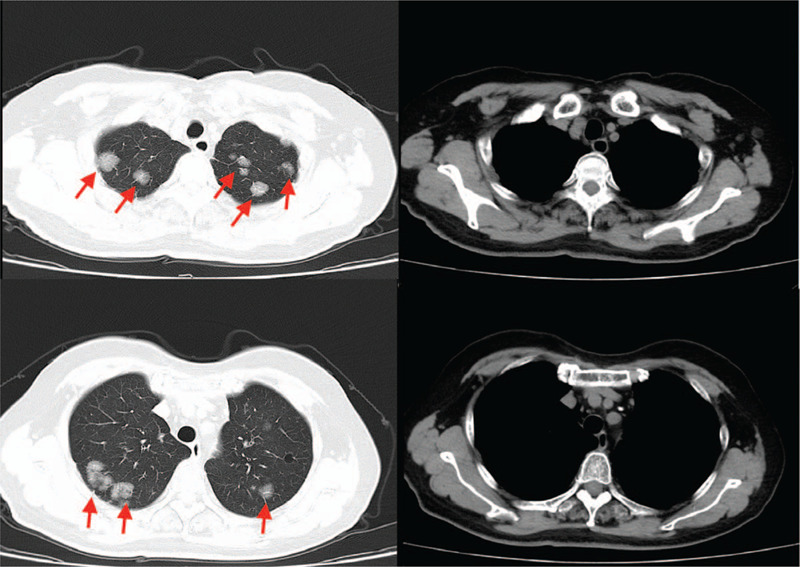
After 1 month of onset, chest CT shows multiple grinding nodes in both lungs. CT = computed tomography.

**Figure 4:**
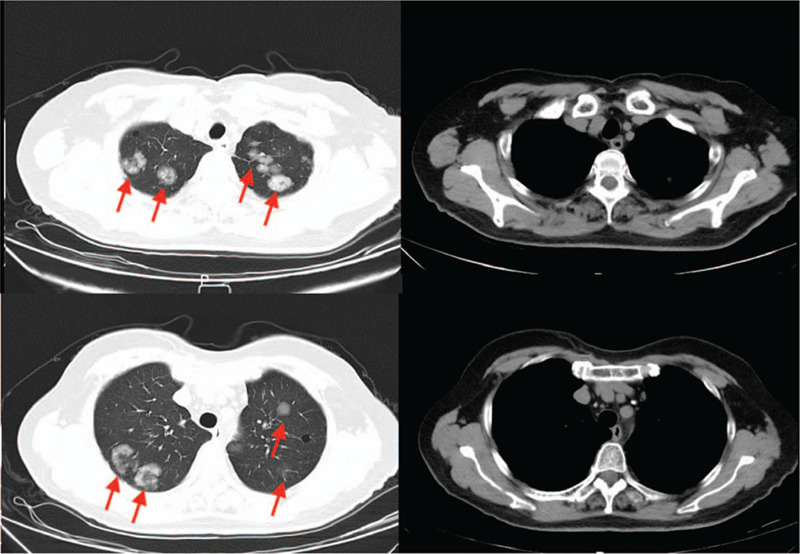
After 2 months of onset, lung nodules increase after the anti-infective treatment.

**Figure 5:**
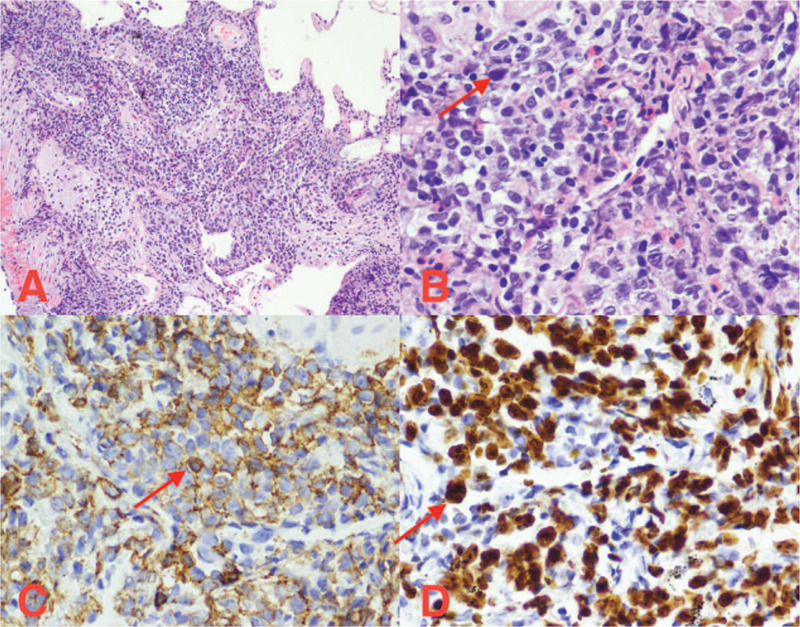
Hematoxylin and eosin (H&E) staining and immunohistochemical staining. (A) Alveolar septal broadens, and tumor cells infiltrate (H&E ×100). (B) The tumor cells are large and rich in the cytoplasm, the karyotype is slightly irregular, and the nucleolus is visible (HE ×400). (C) Immunohistochemical staining for CD20 (×400). (D) Immunohistochemical staining for Ki-67 (×400).

**Figure 6:**
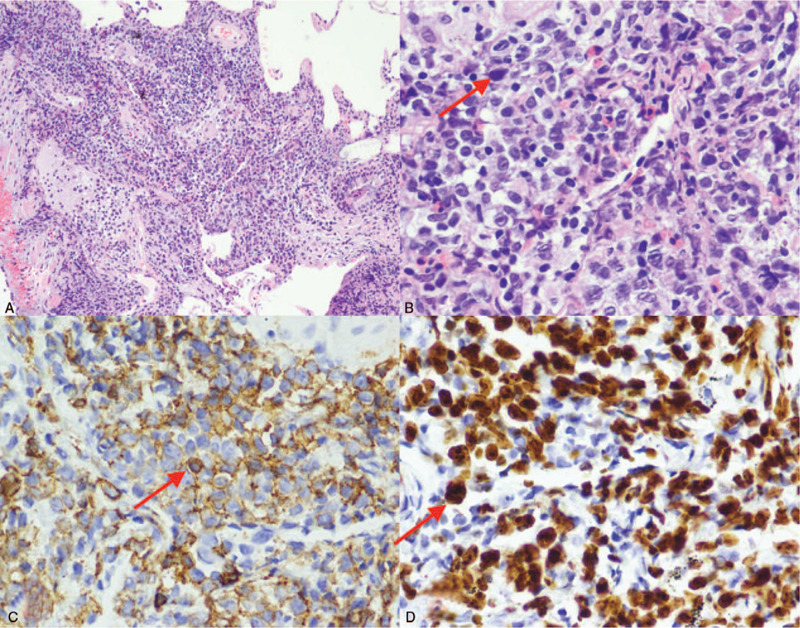
After the third chemotherapy, the nodules are absorbed.

A 60-year-old woman with complaints of having cough was admitted to the Second Affiliated Hospital of Jilin University recently. She had no personal history of allergies and smoking. Also, she had no family history of cancer. There was no obvious cause of cough and sputum 2 months before her admission. Chest computed tomography (CT) (Fig. 1) showed no significant abnormal changes. Her symptoms improved after the oral administration of moxifloxacin. However, symptoms of cough appeared again 1 month later; 18F-PET/CT (Fig. 2) demonstrated multiple lung-grinding glass nodules with a partial metabolic increase, which showed the possibility of an infection. The lung CT showed “multiple nodules” (Fig. 3). She was diagnosed with “pneumonia” and received moxifloxacin plus piperacillin sulbactam in a local hospital. After 2 weeks of treatment, the patient's symptoms improved, and the chest CT (Fig. 4) was re-examined, which showed an increase in the lung nodules. The patient then came to our hospital for further diagnosis and treatment. Physical examination showed the condition to be normal. No palpable lymph nodes and hepatosplenomegaly were found. There was no obvious rale in the bilateral lungs. After admission, the blood routine, erythrocyte sedimentation rate, tuberculosis antibody, related examination of connective tissue disease, tumor marker, sputum culture, acid-fast smear, and exfoliated cell examination showed no obvious abnormalities. Considering her symptoms, including chronic cough, as well as respiratory findings and no history of smoking, the infection remained at high risk in the differential diagnosis. Furthermore, a fiberoptic bronchoscopy needle biopsy was performed. The pathological section (right upper lobe) showed non-Hodgkin's lymphoma with large β cells (Fig. 5). Immunohistochemistry revealed CD20+, BCL-2+, CD10−, BCL6+/−, MUM-1+, CD30+, CyclinD1−, C-myc about 30% cells+, Ki-67 proliferation index >50%, and EBER−. Histopathological staining of the tissue revealed proliferation of large cells (Fig. 5A–D) positive for CD20, CD30, BCL-2, BCL-6, EBER, and MUM-1 and negative for CD10 and cyclin D1. The proliferation marker, Ki67, was highly positive (>50%), indicating a high-grade neoplasm. The patient was finally diagnosed with DLBCL. The patient underwent regular R-CHOP-21 chemotherapy (rituximab, cyclophosphamide, doxorubicin, vincristine, and prednisolone dosing every 21 days), and after the third chemotherapy, the chest CT (Fig. 6) showed that the lesions were reabsorbed. This patient is still under follow-up.

## 3 Discussion

**Figure 7:**
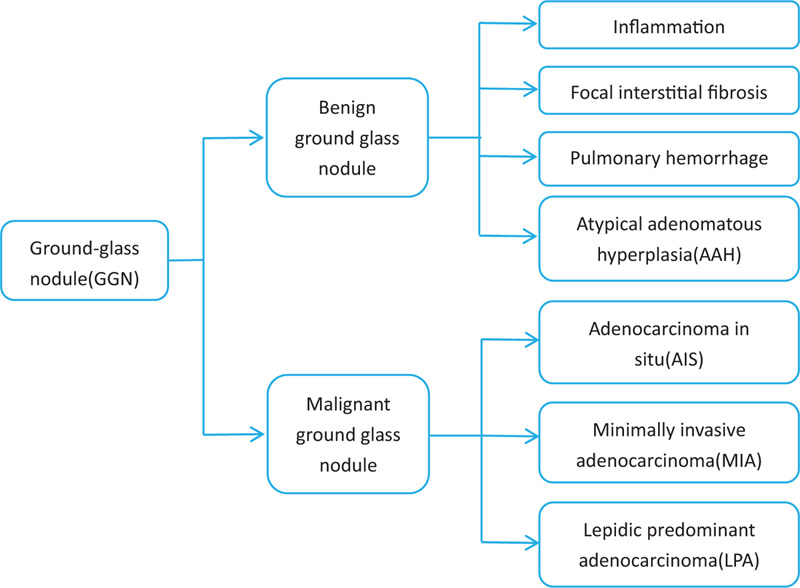
Multiple ground-glass nodule common diseases.

Ground-glass opacity (GGO) refers to the appearance of a thin cloud-like density increase on CT without obscuring the blood vessels and bronchus inside it.^[2]^ As a common but nonspecific sign in the lung, it can be found in many diseases. GGO can be classified as diffuse and focal. The pathological basis of GGO-like lesions is a localized thickening of the alveolar wall, collapse of the alveolar space, reduced air volume in the alveolar space, and filling of fluid or cellular material.^[3]^ The GGO can be benign or malignant. GGN refers to a focal, nodular ground-glass shadow. It is also seen in a variety of diseases (listed in detail in Fig. 7) such as benign lesions, namely focal fibrosis, inflammation, and focal hemorrhage, or adenocarcinoma-based tumor lesions.^[4]^

In this case, the patient was a 60-year-old woman who was admitted to the hospital with symptoms of cough. The patient was healthy in the past. Studies^[5]^ indicate that women have a higher probability of GGNs than men, and the Author considers that the incidence of adenocarcinoma is significantly higher in women than in men. The latest edition of the Fleischner Association guidelines for pulmonary nodules^[6]^ states women are at high risk. Multiple GGNs have a wide age range and can occur in both young and old, but their occurrence in the 56- to 68-year-olds is more common. The distribution of multiple GGNs is irregular and can be located in the same lobe or in a different lung lobe, which is not helpful in assessing whether it is benign or malignant. However, in 2017, the guidelines published by the Fleischner Association^[6]^ pointed out that the distribution of nodules in the upper lobe is considered as one of the risk factors for malignant lesions. There was no obvious abnormality in lung imaging in the early stage of the disease. Two months after the onset of the disease, multiple lobes, and multiple nodules appeared. Combined with a shorter course of disease and the rapid growth of multiple GGNs, it is highly likely to consider benign lesions as inflammation or focal fibrosis. The patient's cough symptoms improved during the anti-infective process, but the imaging changes aggravated, suggesting that the patient had a disease other than what we expected. She was eventually diagnosed with DLBCL by a bronchoscopy lung biopsy.

We found that the lung is the most frequently invaded organ by lymphoma, accounting for 25% to 40% of lymphoma cases,^[7]^ but lymphoma originating in the lungs is uncommon. PPL is defined as an abnormal proliferation of lymphoid tissue in the lung parenchyma or bronchi, with no evidence of extrapulmonary lesions at the time of onset or within 3 months of diagnosis. The most common type of PPL is MALT, which accounts for approximately 70% to 90% of the cases. Less common β-cell lymphomas include DLBCL, lymphomatoid granulomatosis (LyG), plasmacytoma, and other small lymphocytic lymphomas.^[8]^ Among them, DLBCL constitutes 1% of the cases of primary lymphoma of the lung, accounting for 0.4% of all the lymphomas.^[9]^ The clinical features of PPL are not specific, and lung DLBCL can be seen in immunodeficient patients having a vascular collagen disease with or without potential pulmonary fibrosis, patients having AIDS, and patients taking cyclosporine after transplantation. Although the mechanisms of the increased incidence of lymphoproliferative diseases in immunodeficient patients are not fully understood, deterioration in immunoregulation, chronic antigenic stimulation, and tumor suppressor system dysregulation are thought to be the main reasons for it.^[10]^

DLBCL is usually symptomatic, with difficulty in breathing, fever, sweating, and weight loss. For the diagnosis of PPL, these symptoms are nonspecific and contribute very little. In this paper, the patient presented with only repeated cough symptoms.

Under the radiologic findings, PPL has no specificity in lung imaging. Niu et al^[11]^ showed differences in radiological features between PPL and secondary pulmonary lymphoma (SPL). PPL was mainly manifested with lung masses, whereas SPL mainly showed pleural involvement and mediastinal and hilar lymph node enlargement. Radiological manifestations of PPL can be divided into the following 4 modes: nodular, pneumonia or alveolar, bronchial or lymphatic, and miliary nodules.^[12]^ There may be ≥2 types in the same patient. The most common lung pattern lymphoma is the nodule. The most common finding of PPL is the multiple bilateral pulmonary nodule air bronchography.^[13]^ The key features of PPDLBCL's CT are single or multiple solid pulmonary nodules or masses, cavitation, and mediastinal lymph node enlargement.^[14]^ In this case, the patient's lungs showed rare multiple ground-glass-like nodule changes, which were rapidly growing. There have been only few reports in the past regarding the role of 18F-FDG PET/CT in patients with PPL. In our case, the PET–CT showed a part of GNNs with increased fluoro-deoxy-glucose uptake. In a report by Madan et al,^[15]^ the authors showed intense FDG uptake in bilateral cavitary pulmonary nodules, which were histopathologically diagnosed as PPDLBCL. PET-CT can show the distribution of fluoro-deoxy-glucose in the lung mass, and the rest of the body is within normal limits or increasing. After the treatment, there is sometimes a decline in the distribution of fluoro-deoxy-glucose of the lesions. Therefore, a PET/CT study may be helpful in the initial diagnosis and staging of pulmonary lymphoma,^[11]^ and it may be a modality in the management (evaluation of therapy response) of patients with PPL, especially under the DLBCL type.^[16]^ However, the rate of misdiagnosing pulmonary lymphoma is high, and diagnosis must rely on lung tissue biopsy and immunohistochemistry.

PPDLBCL is also considered to be a poor prognosis (5-year survival rate of 0%–60%),^[17,18]^ progressing rapidly, and prone to recurrence. However, Neri et al^[19]^ reported that the use of the CHOP regimen to treat PPDLBCL resulted in a patient-free survival rate of over 90% and a 10-year overall survival rate of over 92%. At present, the patient has received regular chemotherapy for 5 months. During the follow-up, the patient's lung imaging showed that the lesions had been absorbed, and assessment continued.

In the past, we found that CT examination of PPDLBCL showed patchy or flocculent shadows, isolated or multifocal nodules, or masses in single or double lungs. Multiple ground-glass-like nodules in lungs were more common in lung adenocarcinoma. PPDLBCL, with the main manifestation of multiple GGNs, has rarely been reported. This study shows that spotting multiple GGNs in the lungs is a clear indication of the presence of PPDLBCL. The imaging findings of patients with PPDLBCL are complex and diverse, and their clinical manifestations are nonspecific, which often led to misdiagnosis or a delay in diagnosis. After a clear diagnosis in the early stage of PPDLBCL, the corresponding surgical treatment or chemotherapy can be administered. PPL generally carries a poor prognosis, partly because early diagnosis is not possible. Therefore, raising awareness for PPDLBCL is essential for a timely prognosis.

## Author contributions

**Conceptualization:** Qi Wang, He Yan, Rangrang Wang, Zhenzhong Su, Jie Zhang.

**Data curation:** Qi Wang, He Yan, Rangrang Wang, Wei Li, Yanling Xu, Jie Zhang.

**Formal analysis:** Qi Wang.

**Investigation:** Qi Wang, Chunyan Li.

**Resources:** Jie Zhang.

**Writing – original draft:** Qi Wang, He Yan, Rangrang Wang, Chunyan Li, Wei Li, Yanling Xu, Jie Zhang.

**Writing – review & editing:** Qi Wang, Zhenzhong Su, Jie Zhang.
